# Dietary divergence is associated with increased intra-specific competition in a marine predator

**DOI:** 10.1038/s41598-018-25318-7

**Published:** 2018-05-01

**Authors:** Norman Ratcliffe, Stacey Adlard, Gabrielle Stowasser, Rona McGill

**Affiliations:** 1 British Antarctic Survey, High Cross, Madingley Road, Cambridge, CB3 0ET United Kingdom; 2 Life Sciences Mass Spectrometry Facility, Scottish Universities Environmental Research Centre, Rankine Avenue, Scottish Enterprise Technology Park, East Kilbride, G75 0QF United Kingdom

## Abstract

Optimal foraging theory predicts that when food is plentiful all individuals should take a small range of preferred prey types, but as competition increases less preferred prey will be included in the diet. This dietary switching may not be uniform among individuals, which produces discrete dietary clusters. We tested this hypothesis for gentoo penguins at Bird Island, South Georgia, using stable isotope analysis and biologging. Competition, in the form of the density of foraging dives, increased markedly from incubation to chick-rearing owing to increased foraging effort. Birds responded behaviourally by exploiting a greater portion of the available foraging radius and increasing dive depths. Dietary niche width doubled and two discrete dietary clusters appeared; one comprising birds that consumed mostly krill and another that ate a greater proportion of demersal fish. There were no differences in morphology between the dietary classes, but birds in the fish class had a tendency to dive deeper, which suggests a behavioural basis for specialization. Our findings are consistent with the hypothesis that intra-specific competition expands the population’s dietary niche width and drives divergence in diets among individuals.

## Introduction

Optimal foraging theory predicts that, when resources are abundant, animals should select a small number of preferred prey types that provide the highest nutritional return for time and energy expended^1,2^. As the availability of these prey declines, alternative sub-optimal prey should be incorporated into the diet such that individual and population niche widths expand^2^. This dietary niche expansion is expected to occur equally across individuals, such that both population and individual niche widths become broader^1^. However, evidence is emerging that individuals may not exhibit uniform dietary responses to reductions in the availability of preferred prey, such that they diverge into discrete dietary clusters^3^. The decision to join a cluster may be driven by morphology^4^, social dominance^5^ or behaviour^6^ which result in the rankings in the optimality of prey types differing among individuals^7,8^.

Intraspecific competition has the potential to reduce the abundance or accessibility of preferred prey types and hence produce increased dietary variation, both among individuals and across the population^7^. Increases in population niche width and individual specialization in response to elevated intraspecific competition have been convincingly demonstrated in experiments that manipulated the densities of lacustrine fish in enclosures^3,9,10^. In wild populations, studying the interplay between competition and diets depends upon correlative studies where per-capita availability of food varies through space^11^ or time^6,12^. Potential confounding arises where increased diversity of prey types gives rise to higher predator densities, such that variability in niche width and individual specialization might arise from an “ecological opportunity” to exploit different prey types^7^ rather than competition. Information on spatial or temporal variation in prey availability is required to disentangle these processes which can be challenging to obtain, particularly in the marine environment.

An alternative approach is to examine the diets of colonial central place foraging birds, which all share an equal opportunity to feed over the same spatial area, such that variability among individuals must arise from different foraging strategies rather than differences in spatio-temporal availability of resources^13^. The occurrence of large numbers of predators foraging within a maximum radius from the central place often results in high levels of intra-specific competition in the form of interference or prey depletion^14^. Evidence for this comes from animals breeding at larger colonies tending to have greater foraging ranges^15,16^, higher energy expenditure^17^ and lower adult condition or chick growth rates^18^ compared to smaller ones. Abrupt increases in the intensity of intra-specific competition often occurs at hatching when parents are obliged to increase foraging effort to meet the demands of the growing chick^19^, which comprises a natural experiment to test the effects of competition upon dietary niche widths.

Caution is required when attributing changes in diet at hatching to increased competition as other aspects of foraging ecology of seabirds may change simultaneously. The majority of seabird species reduce their foraging range in order to provide their chick with the frequent meals required to sustain growth and survival, which alters levels of competition and affects the accessibility of offshore vs. inshore prey types^20^. Diets may also switch at hatching owing to parents provisioning chicks with different prey to that upon which they feed themselves: possible mechanisms include parents ingesting small prey whilst carrying larger items to chicks in their bills^21^, or parents alternating long self-feeding trips with short chick-feeding trips^22^. Studies seeking to isolate the effects of competition upon diet therefore need to be directed at species that have consistently short foraging ranges throughout the breeding season and feed their chicks by regurgitation.

Gentoo penguins *Pygoscelis papua* represent an ideal model for the study of the effects of competition upon dietary niche breadth and intra-population diet variability. Like all penguins they feed chicks by regurgitation but, unusually, they have consistently short, inshore foraging ranges throughout the year^23^. Compared to congeners with greater foraging ranges, gentoo penguins have small colony sizes^24^ and are more susceptible to breeding failure during years of low prey availability^25^, which are indicative of high levels of density-dependence that arises from intra-specific competition for food. They are dietary generalists at the population level, feeding on a mixture of crustaceans, fish and occasionally cephalopods and typically have broader dietary niches than other penguin species breeding at the same location^23^. Prey preference shows striking spatiotemporal variability at regional, local, annual and seasonal scales in response to the availability of different prey species^23,26^. Despite their high trophic plasticity, the diets of individuals sampled at the same site and time are often dominated by different prey types^27^ and these individual preferences may show consistency within and across breeding seasons^28–30^, suggesting gentoo penguins form generalist populations of specialized individuals. A study of gentoo penguins on the Kerguelen Islands found that trip range and dive depths increase with body mass of individuals, which suggests a morphological basis for such specialization^30^.

This paper examines the changes in diet and foraging behaviour in a colony of gentoo penguins at Bird Island, South Georgia, across the incubation and chick-rearing stages. Changes in diet in terms of the proportion of crustaceans vs. fish are inferred from nitrogen stable isotope ratios, while foraging behaviour is described by tracking birds with GPS and time-depth recorders (TDR). We use this information to examine support for the following hypotheses: (H1) at-sea density and hence intra-specific competition following hatching will increase abruptly owing to birds spending more time at sea without increasing foraging ranges; (H2) birds will reduce this competition by diving deeper to expand their spatial niche width in the vertical dimension; (H3) the population dietary niche width will broaden and discrete dietary clusters of individuals will be formed and (H4) these clusters will be related to differences in individual morphology.

## Results

### Sample sizes

The study resulted in 53 gentoo penguins being equipped with TDRs; one was initialised incorrectly and two more did not go to sea before recapture, resulting in 50 TDR datasets (25 in each stage) which recorded a total of 217 foraging trips and 23,092 dives, all of which were used to assess variation in time budgets and dive depths. Blood could not be obtained from two birds despite attempts on both flippers. Blood congealed before centrifuging in 16 cases and did not separate into fractions, resulting in a sample of 32 birds (16 in each stage) for which plasma was available. GPS tags were deployed on 30 of the TDR-equipped birds and 21 (9 and 12 during incubation and guard, respectively) were recovered along with dive data (producing a total of 84 trips during which 10,901 dives were made). Three failures were caused by the problems mentioned above for the TDRs, and an additional six by malfunctions or flooding of the TrackTags.

### Time budgets

**Figure 1:**
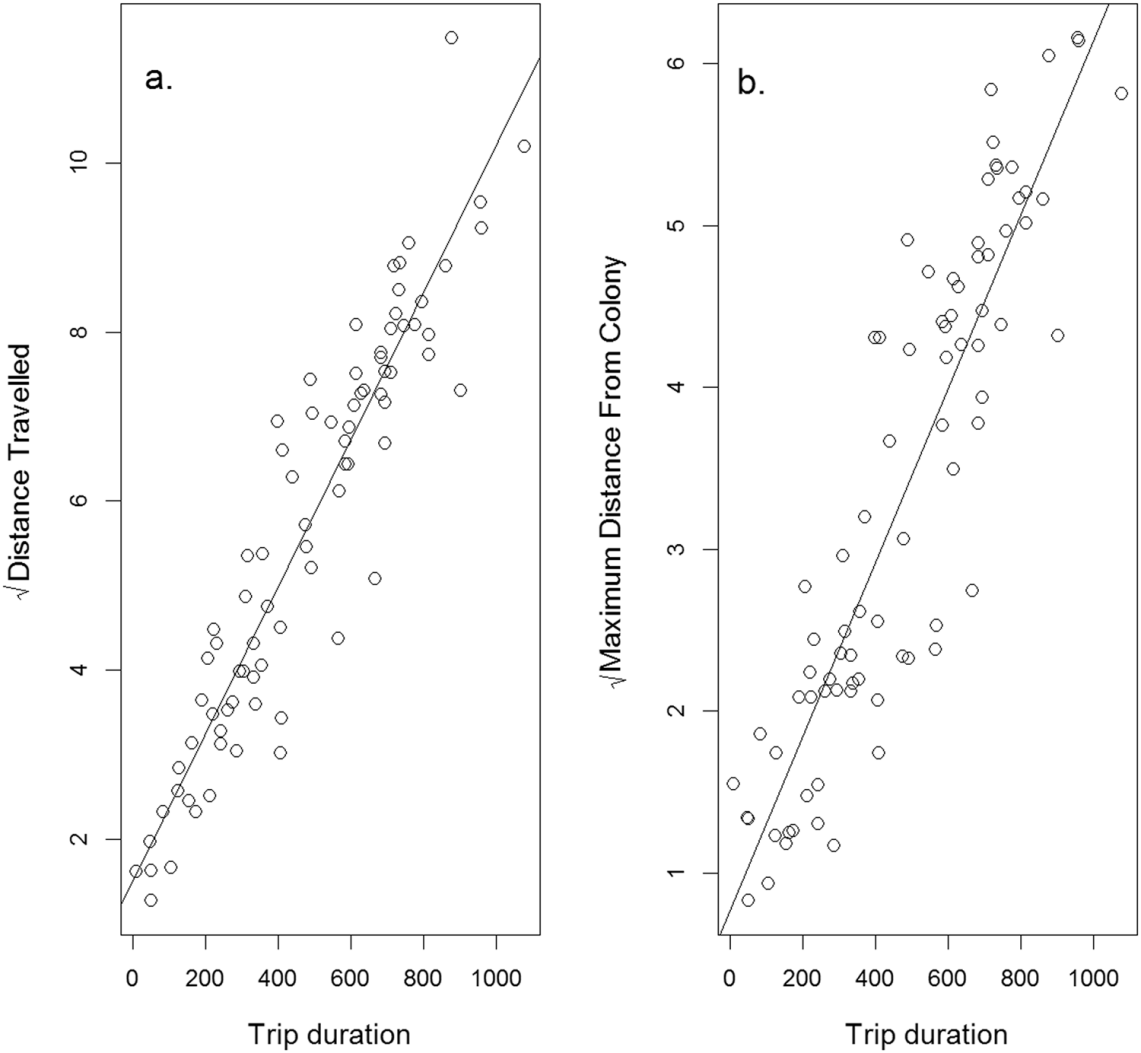
The relationship between (**a**) the square root of the total distance travelled during a trip (km) and (**b**) the square root of the maximum distance reached from the colony (km) in relation to the trip duration (mins). Distance travelled (D) increased with trip duration (t) according to the equation: D = 1.52 + 0.009 √t, while maximum distance from the colony (M) increased with trip duration according to the equation: M = 0.77 + 0.005 √t. The relationships are highly significant (distance travelled: F = 544.9, df = 1, P < 0.0001; maximum distance: F = 342.1, df = 1, P < 0.0001).

There were no significant effects of bird weight (LR = 1.29, df = 1, p = 0.27) or being equipped with a GPS logger (LR = 1.09, df = 1, p = 0.22) on trip durations. The duration of foraging trips was significantly longer during chick-rearing than incubation (LR = 12.19, df = 1, p < 0.0001). Trips during incubation lasted for 291 ± 0.91 (SE) minutes and those during chick rearing were 25 ± 1.08 minutes longer. The random individual effect accounted for 33.5% of the variation remaining once the fixed effects were explained. Based on relationships between travel time and distance metrics (Fig. 1), the average distance travelled during a trip was 16.40 ± 0.01 km during incubation and increased by 3.01 ± 0.03 km during chick-rearing, while the average maximum distance reached from the colony during a trip was 5.40 ± 0.01 km during incubation and increased by 0.82 ± 0.02 km during chick rearing. The greatest distance reached from the colony across all birds and trips, based on observed GPS locations, was 26.7 km during incubation and 37.8 km during chick-rearing.

There was no significant effect of bird weight (LR = 0.01, df = 1, p = 0.97) or being equipped with a GPS logger (LR = 0.13, df = 1, p = 0.67) on the proportion of time at sea, but stage of the breeding season had a highly significant effect (LR = 35.97, df = 1, p < 0.0001). The proportion of time spent at sea was 0.162 ± 0.02 during incubation but more than doubled to 0.360 ± 0.03 during chick-rearing. These values result in daily energy expenditures of 405 kJ day kg^−1^ during incubation, and 483 kJ day kg^−1^ during chick-rearing: a 19% increase. The rate of diving whilst at sea differed significantly among stages of the season (LR = 84.82, df = 1, p < 0.0001), being higher during incubation (15.39 ± 1.01 dives per hour) than during chick-rearing (13.62 ± 1.01).

### Spatial variation in foraging activity

There were no significant effects of bird weight (LR = 1.44, df = 1, p = 0.28) or being equipped with a GPS unit (LR = 1.45, df = 1, p = 0.26) upon dive depths. The effect of the stage of the breeding season on the mean and variation in dive depth was highly significant (LR = 3296.14, df = 2, p < 0.0001). Dives were on average 36.4 m ± 2.13 deep during incubation and 17.7 m ± 3.09 deeper and 1.8 times more variable during chick rearing. The autocorrelation coefficient for successive dives within trips was 0.47. The random individual effect accounted for 10.0% of the variation while that of trip accounted for 17.6%. The deepest dive recorded was 173 m.

**Figure 2:**
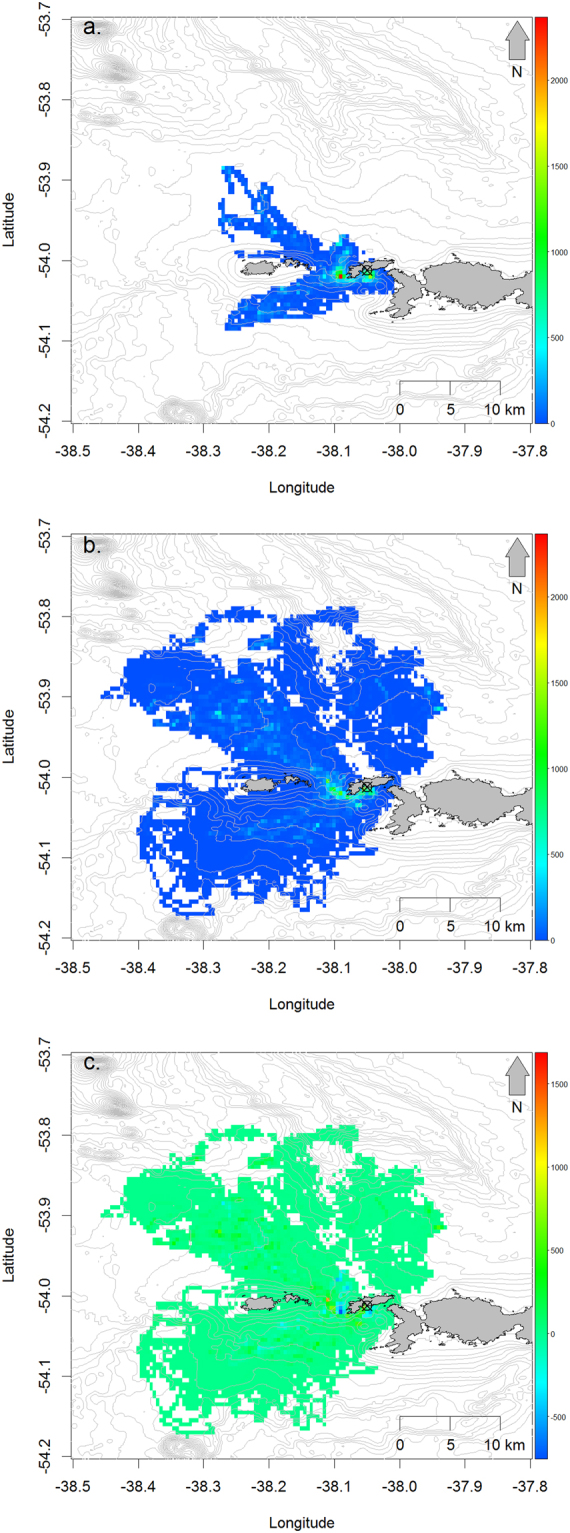
Number foraging dives per day within 0.005 decimal degrees grid cells by gentoo penguins from the Landing Beach and Square Pond colonies during (**a**) incubation, (**b**) chick-rearing and (**c**) the difference between the two (chick-rearing minus incubation densities). Maps were created by the authors using R version 3.4.2 (R Core Team. R: *A language and Environment for Statistical Computing*. R Foundation for Statistical Computing, Vienna, Austria. https://www.R-project.org (2017)).

The spatial distribution and density of foraging dives both increased from incubation to chick rearing. During incubation, dives occupied an area of 137 km^2^ and were concentrated on inshore waters to the immediate south and west of Bird Island and in two prongs to the north and south of the Willis Islands (Fig. 2a). During chick rearing, these areas were again used most intensively, but the total area used increased five-fold to 702 km^2^ and extended across a wider portion of the available radius around the island (Fig. 2b). Despite the expansion in the foraging area, the average foraging intensity during chick-rearing within the area used during incubation still increased from 60.9 to 76.6 dives per 0.005° grid square (Fig. 2c). The exceptions were those cells over the shallows around the south and west coasts of Bird Island and the reefs to the northwest, which were used less during chick-rearing compared to incubation (Fig. 2c).

### Diet

**Figure 3:**
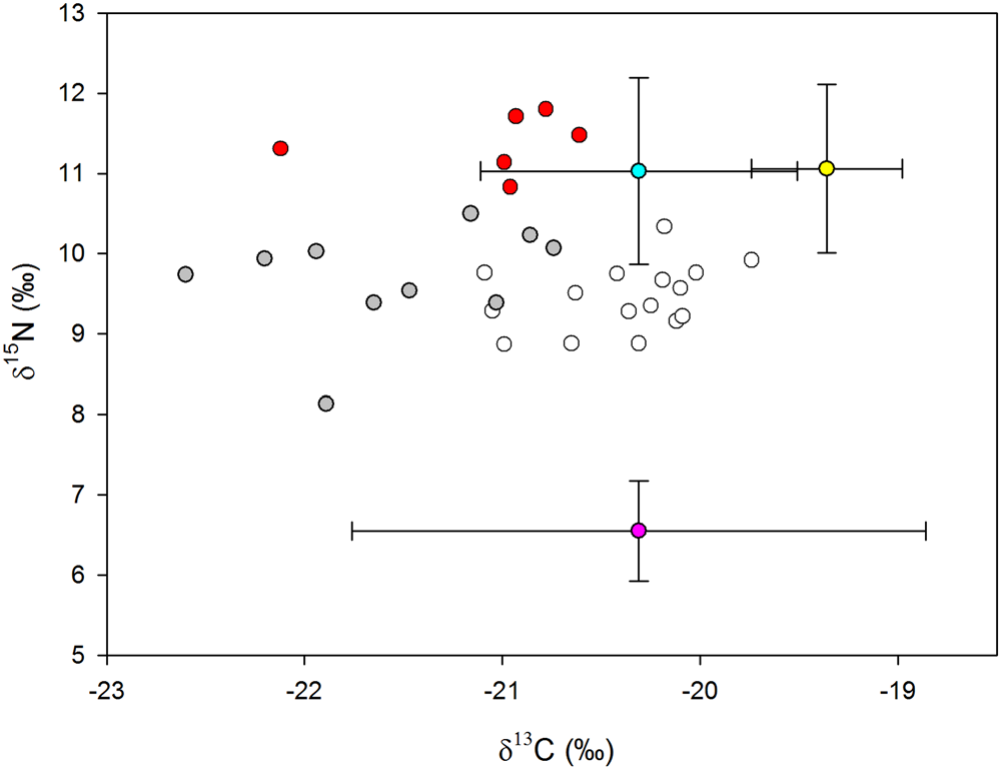
Stable isotope ratios (‰) of gentoo penguins at Bird Island, South Georgia, and their prey. Symbol fills represent gentoo incubation (gray), gentoo chick-rearing diet Class 1 (white), gentoo chick-rearing diet Class 2 (red), Antarctic krill (pink), mackerel icefish (blue) and painted notie (yellow). The bidirectional error bars of the prey values represent 1 SD.

The stable isotope ratios for gentoo penguin plasma during incubation and chick-rearing and of different prey types are shown in Table 1 and Fig. 3. Clear differences were evident in the δ^15^N ratios of fish and krill, while δ^13^C of krill was very variable and overlapped that of fish. Painted noties *Lepidonotothen larseni* had somewhat higher δ^13^C ratios than mackerel icefish *Champsocephalus gunnari* although there was some overlap between them. Gentoo penguins had similar δ^15^N ratios to the fish species while that of δ^13^C was variable, tending to be higher during chick-rearing than during incubation (Fig. 1).

**Table 1:** Stable isotope ratio in plasma of gentoo penguins and in homogenised tissues of their prey (means ± 1 SD).

Sample	δ^15^N	δ^13^C	N
Gentoo penguin incubation	9.41 ± 0.56	−20.08 ± 0.44	16
Gentoo penguin chick-rearing	10.19 ± 0.83	−20.24 ± 0.83	16
Antarctic krill	6.55 ± 0.625	−20.31 ± 1.45	20
Mackerel Icefish	11.03 ± 1.16	−20.31 ± 0.80	9
Painted Notie	11.06 ± 1.05	−19.36 ± 0.38	5

The effect of stage on the mean and variability of δ^15^N was highly significant (LR = 18.9, df = 1, p = 0.0001). The model estimated that δ^15^N was 9.41‰ ± 0.13 during incubation and was 0.78‰ ± 0.21 higher and 2.08 times more variable during chick rearing. This represents an increase in the trophic level at which birds were feeding and a doubling of the dietary niche width. The Gaussian finite mixing model found support for two distinct classes in the δ^15^N data; Class 1 comprising 26 samples with a mean of 9.54‰ ± 0.49 and Class 2 comprising six samples with a mean of 11.31‰ ± 0.49. All of the six samples in Class 2 were collected during the chick rearing stage and so comprised 38% of birds sampled in this period (Fig. 3).

The stable isotope mixing model showed that the proportion of crustaceans in the diet was higher during incubation (0.93; 95% credible intervals = 0.87, (1) than during chick-rearing (0.75; 0.63, 0.87). The proportion of crustaceans in the diet based on stomach content analysis during chick rearing in February 2010 was 0.62, which is at the mixing model’s 95% lower credible interval. When samples were grouped according to the classifications by the Gaussian finite mixing model the proportion of crustaceans in the diet of Class 1 birds was higher (0.92; 0.86, 0.99) than that of Class 2 birds (0.53; 0.37, 0.69).

### Relationships between diet class, behaviour and morphology

Diet class had a significant effect upon dive depths (LR = 6.822, df = 1, p = 0.006), with members of the fish class on average diving 10.68 m ± 4.04 deeper than those of the crustacean class. Variance components analysis showed that diet class explained 34% of the variability in dive depths among individuals but a mere 3% of the combined random and residual variation. There were no differences in trip durations (LR = 0.14, df = 1, p = 0.71), body mass (LR = 3.19, df = 1, p = 0.08) or bill dimensions (LR = 0.57, df = 1, p = 0.4) between the two diet classes.

## Discussion

Tracking of animals with devices has proliferated in the past two decades, and studies need to test the effects of equipping birds with tags for both ethical reasons and to assess bias in the data collected. The effects of devices on behaviour typically increase with weight and drag^31^, so the experimental deployment of minute TDRs or accelerometers offers a powerful means of testing the effects of larger tracking devices on behaviour whilst animals are unobservable at sea^32,33^. Using this approach, we found no support for the hypothesis that GPS devices affected any of the behavioural variables studied, which provides confidence that the parameters presented here are representative of those for unequipped birds in the population.

The stable isotope mixing model estimated a somewhat higher proportion of crustaceans in the diet than did analysis of stomach contents, although these were sampled later in the chick rearing season than the blood samples such that the differences might be due to temporal changes in diet rather than methodology. Other studies of penguin diets have found that stable isotopes provide comparable estimates of diet composition to stomach contents^34,35^, so we surmise that the diet composition inferred from our isotope mixing model is broadly accurate.

Parent gentoo penguins have to increase their foraging effort to meet the food demands of their chicks after hatching, and achieve this by increasing the frequency of foraging trips whilst only increasing trip durations to a modest degree^19^. Many seabird species extend foraging ranges to compensate for high conspecific foraging densities, but gentoo penguins are obligate inshore foragers^23^ and so their at-sea densities are prone to increase markedly whenever foraging effort increases. In our study, birds attempted to reduce at-sea densities during chick rearing by foraging over a greater proportion of their available foraging radius, but densities, and hence intra-specific competition, increased across almost all cells despite this. The only cells in which density declined were those used most intensively during incubation, suggesting these might have been avoided during chick-rearing due to previous depletion of prey.

Adult gentoo penguins eat approximately 1.1 kg of prey per day and chicks require 60 kg over the whole fledging period^36^, so over the course of the 137 day breeding season the 1,933 pairs and 1,604 chicks present on the whole of Bird Island in 2009 would have consumed 680 metric tonnes of prey from an area of approximately 1,000 km^2^. Depletion is therefore plausible in the absence of high rates of prey flux. Interference competition is unlikely to arise as krill avoid predation by diel vertical movement^37,38^, and gentoo penguins diving abilities exceed the water depth across most of their foraging range around Bird Island. This contrasts with studies of species with more offshore distributions where interference was believed to be the key driver of intra-specific competition^16,39^. In concert, these findings are consistent with an increase in at-sea density, intra-specific competition and possibly prey depletion occurring after hatching, which provides support for Hypothesis 1.

The weak compensation for increased foraging density by increasing the foraging range would create a strong selective pressure for niche expansion along other axes. The mean dive depth and variability of dive depths increased during hatching compared to incubation, which indicates that birds were feeding across a wider range of the water column, thus providing support for Hypothesis 2. Similarly, thick-billed murres *Uria lomvia* dived deeper during chick rearing than during incubation^40^ and as prey was depleted through the course of the chick-rearing period^39^. Deeper diving has the potential to either reduce foraging densities in the vertical axis or allow access to alternative deeper-dwelling prey species^41,42^, either of which would reduce intra-specific competition. However, deeper dives are less efficient owing to time spent commuting from the surface and in post-dive recovery, so the benefits of performing them in terms of prey intake have to be sufficient to outweigh these costs^43^.

The dietary niche width of gentoo penguins doubled during chick-rearing compared to incubation due to a switch to fish. Long-term monitoring shows gentoo penguins switch increasingly to mackerel icefish as krill availability declines^44^, and that breeding success is higher in years when diet is krill-dominated^27^. This indicates that krill is the preferred prey and fish are only taken as an alternative when its availability declines. Optimal foraging theory suggests that animals should feed on preferred prey items until intra-specific competition reduces their availability, after which they should switch to less preferred prey types^1,2^. The finding that fish only became important in the diet after hatching when intra-specific competition increased is consistent with this theory. The wider population niche was due to some individuals switching to fish, rather than all birds adopting mixed diets, which produced two distinct dietary clusters comprising krill and fish specialists and provides support for Hypothesis 3. The individual clusters indicates that dietary niche did not merely broaden in order to meet specific nutritional requirements of chicks, otherwise all individuals would be expected to have switched diets in a similar manner post-hatching. Intra-specific competition has also been found to produce dietary clusters of individuals in sticklebacks *Gasterosteus aculeatus*^3^ and food limitation results in greater dietary diversification among individual sea otters *Enhydra lutris*^11^.

Two alternative explanations for the observed changes in diets need to be explored before drawing conclusions regarding the effects of competition. The first is that of “ecological opportunity” in which different prey species become accessible across different stages of the season^7^. Changes in trip durations among breeding stages can affect diets via altered accessibility of inshore vs. offshore prey fields in some seabird species^20^, but this does not apply to gentoo penguins as they forage inshore throughout the year. It is also possible that a decline in the abundance of krill, or increase in that of fish, occurred within the gentoo penguin’s foraging radius and happened to coincide with hatching. This might occur through advection, but flux of krill in currents is low in the inshore areas to the west of South Georgia^45^, while fish of the species and age classes taken by gentoo penguins are resident in shallow shelf waters over reefs and kelp beds^46,47^. Wider-scale declines in krill stocks might also explain the observed patterns, but observations of predator diet and demography^27^ are consistent with a recruitment event occurring during 2009 which would have resulted in krill stocks around South Georgia increasing rather than declining.

The second possible explanation is that inter-specific competition might reduce krill stocks and cause dietary divergence^7^, since gentoo penguins share Bird Island with large colonies of macaroni penguins *Eudyptes chrysolophus* and Antarctic fur seals *Arctocephalus gazella* that consume substantial amounts of krill^48^. However, gentoo penguins have unique foraging niches among Antarctic predators which isolate them from high levels of inter-specific competition^49–51^. We conclude that the weight of evidence points to intra-specific competition being the most likely explanation for the observed patterns of dietary switching and divergence by gentoo penguins.

We were unable to quantify individual specialization in the diet data *sensu stricto* as we had no repeated measures of blood samples from individual birds. Nonetheless, we found discrete dietary clusters in the stable isotope ratios that represent specialization over at least the seven-day period during which stable isotope ratios in blood plasma are integrated. Previous studies have found evidence for high variability in isotope ratios among, and consistency within, individual gentoo penguins^28,30^. Modest levels of between-individual variability were also evident in trip durations and dive depths: values were similar to those found on the Kerguelen Islands, although unlike that study we found no relationship between individual variation and body mass^30^. Individual specialization has also been documented in the diets or foraging behaviour of a wide range of air-breathing marine vertebrates^52–54^.

The weight and bill dimensions of birds did not differ among dietary classes suggesting that there was no morphological basis for dietary divergence, which leads us to reject Hypothesis 4. Likewise, there was no association between diet class membership and morphology in thick-billed murres^55^. Dive depths for gentoo penguins in our study averaged 10 m deeper for members of the fish diet class than for those in the crustacean class which points to a behavioural basis of specialization. Associations between foraging behaviour and diet have been documented in other seabird species^56,57^. A previous study of gentoo penguins at Bird Island also found that birds feeding on fish dived deeper than those feeding on crustaceans, and concluded this was a consequence of krill being pelagic and the fish species being benthic^41^. However, this is an oversimplification, since variability in bathymetry and vertical diel movements of both krill and fish can result in substantial overlap in the depths at which they occur^37,46^. This is likely to explain why specialization in fish or krill only explained a small proportion of the overall variation in gentoo penguin dive depths.

In conclusion, it is widely acknowledged that the restricted foraging radii of gentoo penguins leads to them having small colony sizes and a breeding success that is highly sensitive to variations in food supply^27,58^. Our study reveals the behavioural mechanisms underpinning these observations: high foraging effort during chick-rearing depletes prey in their restricted foraging range and birds attempt to compensate for this by increasing the time spent at sea, expanding the portion of the foraging radius they utilise, making deeper dives and diversifying diets. These compensatory mechanisms will be overwhelmed where large colony sizes or environmentally-induced reductions in prey availability produce particularly high levels of intra-specific competition for food, resulting in breeding failure and population regulation.

## Methods

### Study site and fieldwork methods

The study was conducted between Dec 2009 and Feb 2010 at Landing Beach, Bird Island, South Georgia (−54.01°S; −38.05°W). This beach hosts a colony of 120 pairs of gentoo penguins and is a sub-colony of the larger one at Square Pond (270 pairs and 500 m away). Hatching peaked in the first week of January 2010. To place the study season in context, the gentoo penguin numbers on Bird Island were low compared to the previous and subsequent year while breeding success was moderate (BAS, unpublished data). The low numbers are likely to be due to low Antarctic krill (*Euphausia superba*; hereafter krill) stocks in 2008/09 which caused complete breeding failure and elevated adult overwinter mortality^59^. Acoustic stock assessments to the NW of South Georgia showed krill densities were moderate in 2009, but estimates are unavailable for 2008^60^. These patterns are consistent with moderate krill recruitment into South Georgia waters the spring of 2009.

All methods were carried out in accordance with relevant guidelines and regulations, as described in the permits from the Government of South Georgia and the South Sandwich Islands and the BAS Animal Ethics Panel. Birds leaving their nests were captured using a net before being weighed to the nearest 0.1 kg using spring balance (Pesola, Schindellegi, Switzerland) and having their bill length and depth measured to the nearest 0.1 mm using dial callipers. The nest’s contents were recorded to classify the birds as being at the incubation or chick rearing stage at the start of the deployment. All birds were equipped with G5 standard time-depth recorders (TDR; CEFAS Technology Ltd, Lowestoft, UK), which were attached to the feathers of the lower back using two-part epoxy resin and waterproof tape (Tesa, Hamburg, Germany). These devices weigh 2.7 g in air (1.3 g in water) and have a diameter of 8 mm and length of 31 mm. The average mass of equipped birds was 6.3 kg (SD = 0.75) so the additional load of the logger was trivial. We initialised the loggers to start recording both pressure and temperature every second, starting at 04:00 in the morning so they were logging as birds departed the colony to forage at dawn.

To study horizontal movements of birds we additionally equipped a sub-sample of birds with rapid-acquisition GPS loggers (Fastloc 1; Sirtrack Havelock, New Zealand and TrackTag; Navsys, Edinburgh, UK, the latter enclosed in custom housings manufactured by Bangor University, UK). These weighed 120 g (1.9% of bird weight) with dimensions of 11 cm L, 5 cm W and 2 cm D, and featured saltwater switches and sub-second fix acquisition times to enable positions to be estimated during short periods at the surface between dives. We initialised the loggers to collect positions every 3 minutes and synchronised their clocks and start times with those of the TDR loggers.

Equipped birds were released and then recaptured between four and eleven days later, during which time they completed at least two foraging trips. Upon recapture, the devices were removed and the birds were weighed again. A ~1 ml blood sample was then taken from the brachial vein using a hypodermic needle and syringe, which was transferred to an Eppendorf tube and taken immediately to the laboratory (~5 min walk) for centrifuging and decanting into plasma and cell fractions. These samples were then frozen at −20 °C. Birds were sampled only once over the course of the season owing to ethical considerations of subjecting individuals to these relatively intrusive procedures twice in one season.

### Stable isotope analysis

Plasma and cell samples were freeze-dried and subsequently homogenised using an electronic mixer mill (Retsch, MM200). Samples of ~1 mg were weighed into tin capsules and analysed for δ^13^C and δ^15^N ratios. All isotope analyses were carried out at the Scottish Universities Environmental Research Centre, East Kilbride, UK. Analysis was done by continuous-flow isotope ratio mass spectrometer (CF-IRMS) using a Costech Elemental Analyser (EA) and Thermo Finnigan Delta Plus XP Mass Spectrometer. Laboratory standards were analysed for every 10 unknown samples, correcting for any instrument drift. Stable isotope ratios were expressed in δ notation as parts per thousand (‰) deviation from the international standards Vienna PeeDee Belemnite (carbon) and air (atmospheric nitrogen), according to the following equation δ X = [(R _sample_/R _standard_) − 1] × 1000, where X is ^15^N or ^13^C and R is the corresponding ratio ^15^N/^14^N or ^13^C/^12^C. Measurement precision of both δ ^15^N and δ ^13^C was <0.69‰ and 0.50‰ respectively.

### Logger data analysis

Dive and time-budget statistics were extracted from the TDR data using the R package diveMove^61^. The depth data were zero offset corrected to define the sea-surface, dives were identified using a 2 m depth threshold and the maximum depth was extracted for each. The start and ends of trips were identified by inspection of dive and temperature records. This information was used to derive trip durations and the proportion of time spent at sea for each individual.

We used linear mixed effects models, implemented in the R package nlme^62^, to analyse dive depths, trip durations (square root transformed) and proportion of time at sea (arcsine transformed). Explanatory fixed effects tested were stage of the season (incubation or chick-rearing) and bird mass (as a proxy for both allometric and sex effects, since gentoo penguins exhibit sexual size dimorphism^63^). We included a fixed factor of whether the bird was equipped with a GPS logger to test for device effects. Random effects were trip nested within individual for dive depth, individual for trip duration and none for proportion of time spent at sea, reflecting the different hierarchical sampling of each variable. In the case of dive depth, we also fitted a first-order autocorrelation term within trips to account for the fact that sequential dives tended to reach similar depths^64^. We used a model selection process based on backward stepwise deletion and ANOVA tests. Dive rate per hour was modelled in relation to stage of the season as the number of dives per trip using Poisson errors with the natural log of the trip duration (in hours) as an offset^65^. We converted the proportion of time at sea into energy expenditure using equations in Bevan *et al*.^19^ that were derived from gentoo penguins at Bird Island during the summers of 1991/92 and 1992/93.

The locations of dives along the GPS tracks were modelled using the R package CRAWL^66^. This is a random correlated walk model fitted in a state-space framework, allowing interpolation of the path followed between GPS fixes, with uncertainty. We produced 100 possible locations for each dive made by each individual penguin. These were gridded for all birds and trips (at a cell size of 0.005 decimal degrees) to produce a maps of dive densities by sampled birds during incubation and chick rearing. The estimated dive density per cell per day was calculated as the product of the proportion of dives made within a cell, the number of breeding birds in the Landing Beach and Square Pond colonies (pairs multiplied by two), the number of hours they spent at sea per day (the proportion of time at sea multiplied by 24) and the dive rate per hour.

### Stable isotope data analysis

δ^15^N is a proxy for the trophic level at which animals feed^67^. Gentoo penguins at South Georgia mostly feed on Antarctic krill or one- and two-year old mackerel icefish and painted notie^27^ which themselves feed upon krill^47^. The fish are therefore a trophic level higher than crustaceans so δ ^15^N provides information on consumption of these broad dietary components^35^. δ^15^N in penguin blood cells and plasma have different turnover rates of approximately 32 and seven days, respectively^67^. The slow turnover rate in cells would have resulted in chick-stage samples collected within a month of hatching containing isotope ratios from incubation^20^. We therefore focussed on analysis of the plasma samples only.

General least squares (gls) models fitted in the R package nlme were used to test the fixed effects of stage on average δ^15^N ratios and their variance. We used a finite Gaussian mixtures model to examine evidence for discrete dietary clusters in the δ^15^N data, implemented in the R package mclust^68^. This approach has been used previously to identify dietary classes from isotope ratio data in penguins^20^. Relationships between diet class (as identified by the mclust analysis) and behavioural traits were investigated by including class as a fixed factor in the previously identified best-fit models of dive depths and trip durations. Variance components analysis was used to investigate the proportion of the individual variance in behaviour that was explained by diet class^13^. Relationships between diet class and the body mass and bill dimensions (bill length × bill depth) were investigated using gls models with class as an explanatory factor.

The diet composition during each stage, dietary cluster and individual was inferred from δ^15^N using Bayesian mixing models implemented in the R package SIAR^69^. We used a two-source mixing model to estimate the proportion of the diet comprising crustaceans^35^. Prey items were collected from gastric lavage of 38 adult gentoo penguins on Bird Island during Jan and Feb 2010 were sorted into the lowest possible taxonomic order and weighed. The contents expressed as percentage wet mass of crustacean in each stomach sample to allow comparisons with diet composition estimated by the stable isotope mixing model. Sample specimens were collected from stomach-flushed samples collected in July-Sep 2009 (one per bird) were delipidated, freeze dried and milled, and used to estimate the mean and SD of δ^15^N for krill and fish. We used a trophic enrichment factor of 2.7 in the mixing model to adjust for increases in δ ^15^N of prey following ingestion and assimilation by penguins^70^.
